# The Genetics of Pemphigus Vulgaris

**DOI:** 10.3389/fmed.2018.00226

**Published:** 2018-08-14

**Authors:** Dan Vodo, Ofer Sarig, Eli Sprecher

**Affiliations:** ^1^Department of Dermatology, Tel Aviv Sourasky Medical Center, Tel Aviv, Israel; ^2^Department of Human Molecular Genetics and Biochemistry, Sackler Faculty of Medicine, Tel Aviv University, Tel Aviv, Israel

**Keywords:** pemphigus, genetics, autoimmunity, blistering disorders, HLA

## Abstract

Pemphigus vulgaris (PV) is a severe autoimmune blistering disease caused by auto-antibodies (auto-Abs) directed against epithelial desmosomal components and leading to disruption of cell-cell adhesion. The exact mechanisms underlying the disease pathogenesis remain unknown and treatment is still based on immunosuppressive drugs, such as corticosteroids, which are associated with potentially significant side effects. Ethnic susceptibility, familial occurrence, and autoimmune comorbidity, suggest a genetic component to the pathogenesis of the disease, which, if discovered, could advance our understanding of PV pathogenesis and thereby point to novel therapeutic targets for this life-threatening disorder. In this article, we review the evidence for a genetic basis of PV, summarize the different approaches used to investigate susceptibility traits for the disease and describe past and recent discoveries regarding genes associated with PV, most of which belong to the human leukocyte antigen (HLA) locus with limited data regarding association of non-HLA genes with the disease.

## Introduction

Pemphigus is a group of rare, chronic, autoimmune blistering diseases, which affect the skin and mucosal membranes (1). The annual incidence of pemphigus varies among different populations and is estimated to range between 0.75 and 5 new cases per million (1). PV, the most common subtype of the disease, is characterized by ulcerations or flaccid blisters on mucous membranes and on the skin that easily rupture to cause painful, large erosions, which do not easily heal and which, if not properly treated, can lead to serious life-threatening infections and metabolic disturbances. The use of immunosuppressive drugs, which are the mainstay of treatment, have reduced the mortality from the disease to around 10%, though the adverse effects of corticosteroid treatment still cause considerable morbidity (1). PV is traditionally considered to result from the deleterious action of circulating auto-Abs, which are directed against desmosomal components, primarily desmoglein (Dsg) 3 and Dsg1, and lead to loss of keratinocytes cell-cell adhesion within the epidermis, a phenomenon known as acantholysis (2). In recent years, in addition to desmosome destabilization, blister formation in PV was suggested to result from other pathomechanisms that may be involved in PV pathogenesis and include increased secretion of pro-inflammatory mediators, abnormalities in intercellular signaling, activation of apoptosis and activation of specific muscarinic receptors expressed by keratinocytes (3–7).

Numerous studies provide support for a genetic contribution to the pathogenesis of PV as evident from the ethnic clustering of PV, the familial aggregation of the disease and the higher prevalence of additional autoimmune conditions in both PV patients and their family members (8–13). Unfortunately, although multiple attempts to identify susceptibility traits were set forth, our knowledge regarding the genetic basis of PV is far from complete. Discoveries concerning the genetics of PV will improve the understanding of the mechanisms underlying this severe disease, which in turn may point to novel potential therapeutic targets. In this article, we review our current understanding of the genetics of PV. We describe previous attempts at identifying disease susceptibility loci and review the evidence for the contribution of human leukocyte antigen (HLA) and non-HLA genes to PV pathogenesis.

## Evidence for a genetic basis of PV

Evidence for genetic susceptibility to contract a given disease often stems from observational studies showing varying disease prevalence in different populations, familial aggregation, and higher concordance among monozygotic vs. dizygotic twins. Over the years, many such studies have firmly established that susceptibility to PV is to a large extent genetically determined.

First, PV prevalence and incidence are low but differ significantly among diverse ethnic populations as evident by the wide range of the worldwide annual incidence of the disease, estimated to be between 0.75 and 5 new cases per million (1). For example, the disease is between 4- and 10-fold more common in the Jewish population in comparison with other Caucasian populations, with an annual incidence ranging from 15 to 30 cases per million (8). Differences in PV incidence between ethnic groups living in the same environment have also been shown and highlight the contribution of genetic, rather than environmental, factors to PV. An epidemiological study performed in Hartford County, USA, showed a 7.5-fold increase in PV incidence in Jewish adults as compared to the overall adult population (14) and a higher incidence of PV was observed in Turks and Italians living in Germany as compared to native Germans (15). A north-south gradient in PV incidence has also been observed, with PV being more common in lower latitudes, such as in Italy or Tunisia (3 and 6.7 cases per million, respectively) than Northern countries such as France or Finland (1.7 and 0.76 cases per million, respectively). This distribution could be influenced by environmental factors but could also be due to specific genetic factors (8).

In addition to population studies, familial aggregation offers yet another clue for a genetic contribution to PV. Though not common, familial cases of PV have been documented, usually involving a first-degree relative (16–19). Moreover, circulating PV-IgG Abs have been found more frequently in first-degree unaffected relatives of PV patients as compared with healthy controls (9). The prevalence of autoimmune conditions in family members of PV patients was also shown to be significantly increased, supporting an inherited susceptibility for autoimmunity, influenced by genetic factors. Compared to controls, family members of PV patients, most commonly first-degree relatives, exhibited a higher prevalence of type 1 diabetes mellitus (T1DM), autoimmune thyroid disease (AITD), and juvenile rheumatoid arthritis (10, 20).

Supporting a genetic background common to PV and other autoimmune conditions, a cross-sectional study of almost 800 PV patients revealed a significant increase in the prevalence of AITD, rheumatoid arthritis (RA), and T1DM in PV patients, in comparison with the general population (13). Comparable results were obtained in a study of 295 PV patients, showing a higher incidence of hypothyroidism, inflammatory bowel disease, and T1DM (21). In contrast, a study of 1998 PV patients, conducted in Taiwan, did not find an association between PV and RA or AITD but discovered a higher incidence of systemic lupus erythematosus (SLE) in PV patients and demonstrated that female PV patients are more likely to suffer from Sjögren's syndrome as well as from alopecia areata (22).

## Strategies for the identification of genetic factors in PV

PV is a multifactorial disease, in which the risk of an individual to be affected with the disease is dependent on a combination of multiple genetic as well as environmental factors. For polygenic conditions, risk alleles are more probabilistic than deterministic, as a person carrying a high-risk trait can be only mildly prone to develop the disease. Nevertheless, identifying these susceptibility genes is crucial for a better understanding of a disease pathogenesis. One common method to identify genetic variants determining the propensity to develop a complex disease is known as association studies (23), in which the frequencies of genetic variations are compared between individuals with the disease and unaffected controls. When an allele shows a higher frequency in the affected individuals, it is considered to be in association with an increased risk to develop the disease. This association points to a region in which the causative variant lies in close proximity to the disease-associated allele (23). Association studies can be conducted in two ways. First, by using a candidate-gene driven approach, in which the regions chosen to be inspected and genotyped are ones carrying genes that, based on previous knowledge, may possibly be involved in the disease pathogenesis. Second, they can be performed without any prior hypothesis over the entire genome and are then known as genome wide association studies (GWAS).

The use of the candidate-gene approach in PV is mainly focused on genes encoding proteins of relevance to immune dysregulation and autoimmunity, such as target antigens, antigen-processing or presenting molecules, proteins related to lymphocytes function, or structure and cytokines. Over the years, most of the candidate gene-driven case-control studies, aimed at identifying genetic factors in PV, have shown associations between several HLA alleles and PV in specific ethnic groups of PV, as described below. In addition, PV was found to be associated with non-HLA genes at the HLA locus, such as *TNF-*α, *IL-6, IL-10*, and *TAP2* (24–28) as well as with genes encoding pemphigus autoantigens (29, 30). However, these data have not been reproduced in similarly designed studies and in additional populations and conflicting results have been published regarding a possible association between single nucleotide polymorphisms (SNPs) in genes coding for cytokines and PV (24, 26, 31). In recent years, new approaches to identify candidate genes are emerging. Among these, is the use of gene expression analysis to identify genes which are differentially expressed between diseased and healthy individuals and that can be further investigated. Pathway analysis of these selected genes can lead to the identification of additional genetic factors which might be involved in the disease pathogenesis. Using this approach, Dey-Rao et al. have discovered subsets of disease-promoting and disease-preventing genes in a study of 21 PV patients (32) while Sezin et al. identified shared gene signatures between PV and SLE and discovered a possible involvement of the gene *GP9* in PV, which encodes a glycoprotein related to platelet adhesion (33).

In contrast to the candidate-gene strategy, a genome wide approach offers a hypothesis-free study and reveals associations with genes with an unknown relevance to the pathogenesis of the disease (34). The aim of genomic-wide scans is to identify co-segregation of genetic markers, previously consisting of fragment length polymorphisms and tandem repeats and more recently of SNPs, in order to define chromosomal regions containing susceptibility loci. However, in order for the results to reach statistical significance, a genome wide approach requires a large study cohort and until lately, the low prevalence of PV has represented a significant obstacle to GWAS in this disease. Sarig et al. reasoned that performing a GWAS in a genetically homogenous study group, with a relatively high prevalence of PV, such as the Jewish population (8) could help to filter out false positive association signals while uncovering significant associations, even from a relatively low number of participants (35). They performed the first GWAS in PV in a Jewish population and discovered several PV-associated markers, as described below.

While both of these methods have been used over the years in order to identify genetic factors involved in PV, novel study designs, combining the two approaches and utilizing new technologies are emerging. The advantage of utilizing gene expression studies in order to prioritize target candidate genes for SNPs in a GWAS has already been demonstrated (36) and was also implemented in PV, leading to the identification of a number of transcriptional hot spots, harboring several genes with possible involvement in PV (32). The use of next generation sequencing (NGS) in PV, including whole genome sequencing and exome sequencing may also increase the chance to identify novel genetic variants as was performed in other polygenic skin disorders (37) and could also assist in identifying causal variants underlying genome-wide associations (38).

## Association of HLA genes with PV

The majority of studies regarding genetic predisposition to PV have been focused on the association between the disease and genes in the major histocompatibility complex, which is termed HLA in humans. This approximately 4 Mb-long region on chromosome 6p21.3, encodes above 200 genes and has the highest gene density in the human genome (39). The HLA locus is divided into three main regions: the class I region encodes the polymorphic HLA-A, HLA-B, and HLA-C genes, which assist in the presentation of antigenic peptides to cytotoxic T-cells and are expressed ubiquitously; the class II region contains polymorphic HLA-DQ, HLA-DR, and HLA-DP genes that are expressed on antigen-presenting cells (APCs) and assist in peptides display to helper T-cells; and the class III region which consists of multiple important immune system-related genes (for example, *TNF-*α*, C2*, and *C4*) (39). A large number of autoimmune disorders were found to be associated with the HLA region, making HLA associations a hallmark of autoimmunity (40).

**Figure 1:**
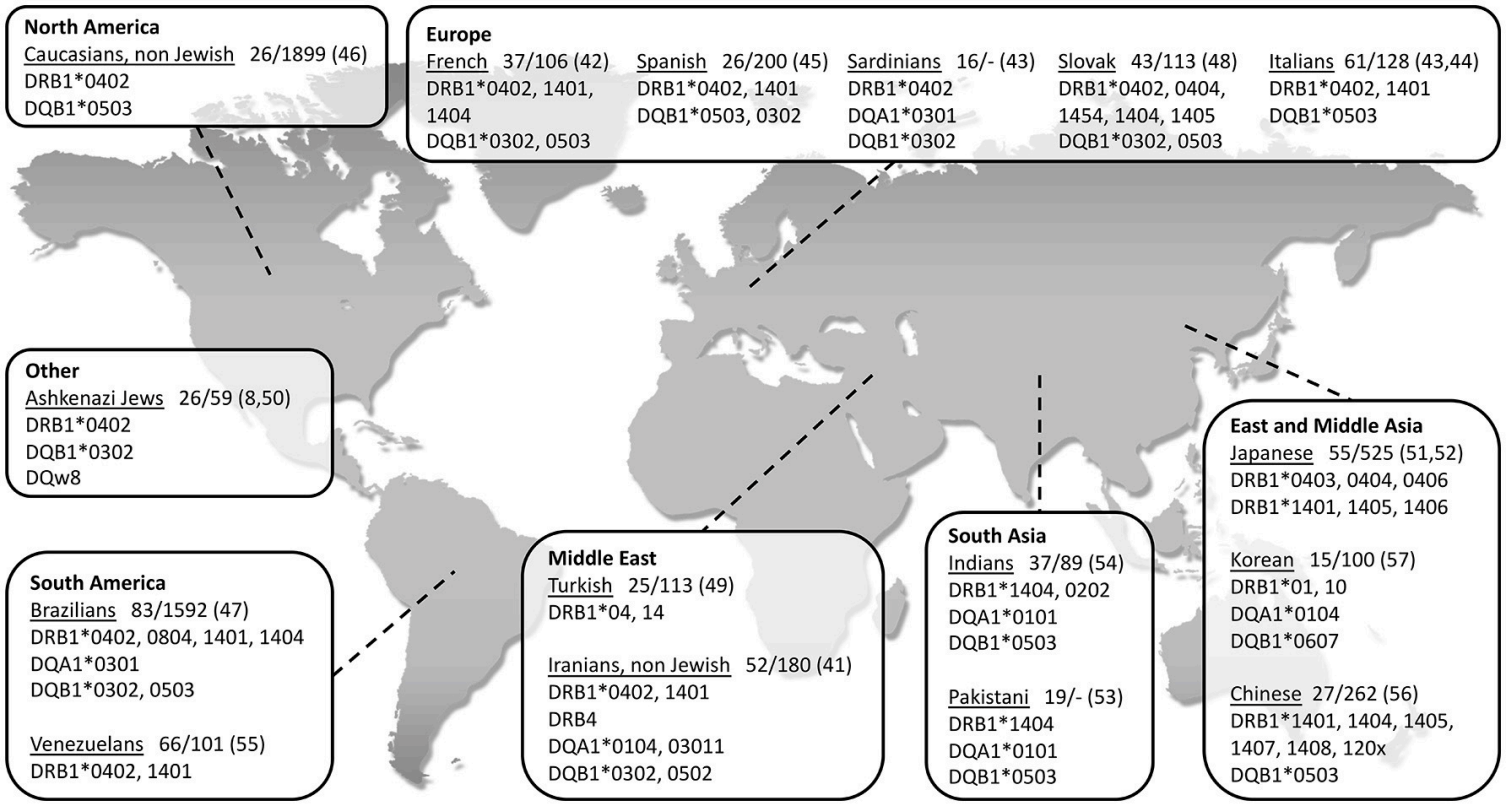
World map showing association of type II HLA genes with PV in different populations, grouped according to geographical regions. The name of each investigated population is followed by: 1. Patients/control (*n*); 2. Reference number in brackets.

So far, the association between PV and HLA class II genes remains the strongest and the most reported. As seen in Figure 1, a vast number of studies have been able to show an association between PV and several class II HLA alleles in specific ethnic groups of PV (8, 41–57). While some of the HLA types are more population specific, there are also ones associated with PV in numerous ethnic groups. The two most common PV-associated alleles are DQB1^*^0503 and DRB1^*^0402, both of which were found to associated with the disease in the Spanish, French, Italian, Slovak, North American and Brazilian populations (42–48) (Figure 1). In the Jewish population, an association was found between PV and several HLA alleles, such as HLA-DRB1^*^0402, and DQB1^*^0302 (8) while HLA-DQB1^*^0503 was found in association with PV in non-Jewish populations (58). A meta-analysis of the correlation between PV and HLA-DRB1 concluded that HLA-DRB1^*^04 and HLA-DRB1^*^14 are indeed statistically significant susceptibility factors for PV along with an additional HLA allele, DRB1^*^08 (59). Interestingly, while HLA-DRB1^*^0402 confers susceptibility to PV, it was found to encode a sequence motif that exerts a protective effect against RA (60).

Over the years, several studies provided evidence regarding association between PV and certain HLA class I alleles. These include HLA-A3, -A26, and -B60 in Han Chinese population (56), HLA-B38, -C12, -B57, and -C15 in the Brazilian population (47, 61), HLA-A10 and -B15 in the Japanese population (62, 63), HLA-B35 and -B44 in the Turkish population (64), HLA-B38 in the Jewish (50) and in the Spanish (45) populations, and HLA-B4402, -C0401, and -C1502 in the Iranian population (65). However, it remains unclear how do HLA class I alleles contribute to PV susceptibility. In addition, several studies have shown an association between PV and non-classic HLA class Ib alleles (HLA-E, -F, and -G). HLA-G polymorphism was found in a significant association with Jewish PV patients (66) while HLA-E, previously demonstrated to play a role in multiple autoimmune conditions (67, 68), was found in association with Caucasian and Ashkenazi Jewish patients and was suggested to be involved in the disruption of immune tolerance in PV (69).

## Association of non-HLA genes with PV

To date, only a limited number of studies have been able to show an association between non-HLA genes and PV (Table 1). Most of these studies have used a candidate gene approach to investigate a possible association between PV and autoimmune-related genes, mainly autoantigens, cytokines, and immunoglobulins known to play a role in PV pathogenesis, such as TNF-α, IL-6, and IL-10 (1, 5, 72, 73). However, the results of these studies have often been conflicting, limited to specific ethnic groups and could not be replicated in similarly designed studies and/or other populations. *TNF-*α was found to be weakly associated to PV in the Slovak population (25) but not in Polish (26) or Argentinian (24) patients while in the Egyptian population only one genotype within the *TNF-*α and *IL-6* genes were found in association with PV (27). A genetic variant within the *IL-10* gene was found in association with PV in Argentinian patients (24) but not in the Slovak population, where only a haplotype inside *IL-10* showed an association to the disease (25). Slomov et al. have shown an association between PV in the Jewish population and the *TAP2* gene, encoding for a protein involved in peptides assembly and transport to HLA class I antigens (28) but this was not reproduced in the Japanese population (74). As for autoantigens, two different haplotypes within the *DSG3* gene were found in association with PV in British and Northern Indian patients, respectively. Interestingly, patients carrying any one of these two risk haplotypes were always found to carry PV-associated HLA class II alleles, suggesting possible additive effects for these two loci (29). A study of 12 PV patients discovered an association between PV and a SNP within the *VH3* gene, encoding part of the variable region of the immunoglobulin heavy chain. However, a sampling error is possible due to the small size of the patient group. Of note, no association was found between PV and the genes encoding for the constant regions of the kappa light chain or heavy chain of the immunoglobulin (70, 75). Tanasilovic et al. reported an association between PV and a SNP within the *CD86* gene (71), encoding for a protein expressed on APCs, which has a role in T-cell activation and IgG4 production by B cells (76). This SNP has the potential to alter CD86 signaling, suggesting that it may have an effect on the production of Dsg3-specific IgG4 Abs, shown to be implicated in PV (77). Using a GWAS in the Jewish population, Sarig et al. has managed to show an association of several genetic variants with PV, including SNPs within the *ST18* gene (35), encoding for a transcription factor (TF) shown to be involved in inflammatory and apoptotic processes (78). Although not showing association with PV in German or Chinese patients, *ST18* was also found to be associated with the disease in the Egyptian population and to be overexpressed in the non-lesional skin of Jewish PV patients (35, 79). A subsequent study showed that the promoter region of *ST18* harbors a PV-associated SNP, which was demonstrated to increase gene transcription. ST18 was additionally shown to stimulate PV serum-induced acantholysis and secretion of key inflammatory molecules, supporting a direct role for ST18 in PV pathogenesis (80). In the Tunisian population, SNPs within the *FOXP3* gene were found in association with the susceptibility and clinical course of Pemphigus Foliaceus (PF), a different subtype of the disease (81). *FOXP3* encodes for a TF with a central role in the development and function of regulatory T (Treg) cells, suggesting it may be involved in the disruption of immune self-tolerance in the disease. Of interest, a significantly reduced number of Treg cells was discovered in the peripheral blood of both PV and PF patients in comparison to controls (82).

**Table 1:** Association of non-HLA genes with PV.

**Gene**	**Role of protein**	**Population**	**Patients/Control (*n*)**	**References**
DSG3	Autoantigen in PV	United Kingdom	62/154	(29)
		India	28/98	(29)
VH3	Part of the variable region of the immunoglobulin heavy chain	French, Italian, and Jewish	12/–	(70)
TNF-α	Inflammatory cytokine	Slovak	34/140	(25)
		Egyptian	51/203	(27)
TAP2	Assembly and transport of peptides to HLA class I antigens	Jewish	37/37	(28)
IL-6	Pro-inflammatory interleukin	Egyptian	51/203	(27)
IL-10	Pro-inflammatory interleukin	Slovak	34/140	(25)
		Argentinian	17/24	(24)
ST18	Transcription factor with roles in the regulation of inflammation and apoptosis	Jewish	100/400 59/285	(35)
		Egyptian	126/246	(35)
CD86	A type I membrane protein which is expressed on antigen presenting cells and provides costimulatory signals necessary for T-cell activation and survival	Serbian	48/486	(71)

## Conclusions

The important role of genetic factors in determining the propensity to develop PV is evident from the numerous epidemiological and association studies reviewed in this article. To date, the majority of studies aimed at identifying susceptibility genes for this complex disease, mainly focused on the HLA locus, showing various associations between PV and HLA alleles. In recent years, new methods, including a genome wide approach, gene expression analysis and NGS, are utilized and new discoveries regarding non-HLA genes involvement in PV are emerging. As the search for the genetic basis of PV continues, our understanding of PV pathomechanism is improving, paving the way for innovative and possibly safer therapeutic approaches.

## Author contributions

All authors listed have made a substantial, direct and intellectual contribution to the work, and approved it for publication.

### Conflict of interest statement

The authors declare that the research was conducted in the absence of any commercial or financial relationships that could be construed as a potential conflict of interest.
